# Gender Differences in Risk Factors for Adolescent Binge Drinking and Implications for Intervention and Prevention

**DOI:** 10.3389/fpsyt.2017.00289

**Published:** 2017-12-22

**Authors:** Allyson L. Dir, Richard L. Bell, Zachary W. Adams, Leslie A. Hulvershorn

**Affiliations:** ^1^Department of Pediatric Adolescent Medicine, Indiana University School of Medicine, Indianapolis, IN, United States; ^2^Department of Psychiatry, Indiana University School of Medicine, Indianapolis, IN, United States

**Keywords:** adolescence, binge drinking, gender, intervention, comorbidity, prevention

## Abstract

Alcohol use, particularly binge drinking (BD), is a major public health concern among adolescents. Recent national data show that the gender gap in alcohol use is lessening, and BD among girls is rising. Considering the increase in BD among adolescent girls, as well as females’ increased risk of experiencing more severe biopsychosocial negative effects and consequences from BD, the current review sought to examine gender differences in risk factors for BD. The review highlights gender differences in (1) developmental-related neurobiological vulnerability to BD, (2) psychiatric comorbidity and risk phenotypes for BD, and (3) social-related risk factors for BD among adolescents, as well as considerations for BD prevention and intervention. Most of the information gleaned thus far has come from preclinical research. However, it is expected that, with recent advances in clinical imaging technology, neurobiological effects observed in lower mammals will be confirmed in humans and *vice versa*. A synthesis of the literature highlights that males and females experience unique neurobiological paths of development, and although there is debate regarding the specific nature of these differences, literature suggests that these differences in turn influence gender differences in psychiatric comorbidity and risk for BD. For one, girls are more susceptible to stress, depression, and other internalizing behaviors and, in turn, these symptoms contribute to their risk for BD. On the other hand, males, given gender differences across the lifespan as well as gender differences in development, are driven by an externalizing phenotype for risk of BD, in part, due to unique paths of neurobiological development that occur across adolescence. With respect to social domains, although social and peer influences are important for both adolescent males and females, there are gender differences. For example, girls may be more sensitive to pressure from peers to fit in and impress others, while male gender role stereotypes regarding BD may be more of a risk factor for boys. Given these unique differences in male and female risk for BD, further research exploring risk factors, as well as tailoring intervention and prevention, is necessary. Although recent research has tailored substance use intervention to target males and females, more literature on gender considerations in treatment for prevention and intervention of BD in particular is warranted.

## Introduction

Binge drinking (BD) is a major public health concern, and adolescents are particularly vulnerable to the biological and social consequences of BD compared to adults (1). Internationally, BD is more prevalent among adolescents aged 15–19 compared to all other adults aged 25 and older (2–6). For example, recent United States national data estimates that 17.7% of high school students (7) and 39% of college students (8) reported BD in the past month, with college students often consuming at least two to three times the definition of BD (9). Rates of BD in Europe and Australia are typically higher than in the U.S. For example, one study of 36 European countries found that 39% of 15- and 16-year-olds reported BD in the past month (10). More importantly, it is well established that an early onset of alcohol use is a strong predictor of future alcohol dependence (11, 12). Significantly, about half of individuals meeting life-time diagnostic criteria for an alcohol use disorder (AUD) do so by the age of 21, with two-thirds meeting criteria by the age of 25 (13–21).

While estimates have traditionally shown higher rates of BD in males, recent national data show that the gender gap in BD is lessening, with a concomitant increase in rates of alcohol use and BD among girls and women (17). In fact, some studies have found that girls are drinking as much, if not more, than their male peers, and girls are also initiating alcohol use earlier and engaging in more binge-like alcohol drinking, while these changes have not been seen among boys in recent decades (7, 17, 22–24). Due to these increasing rates of alcohol initiation and problems among girls, some efforts have been made to create gender-informed interventions and preventions in order to better target adolescent girls (24, 25).

It is also well known that girls are more vulnerable to the negative consequences from alcohol use and BD compared to boys. Across the lifespan, females are more likely to experience alcohol-related health problems at lower drinking rates compared to males, and are also more likely to experience more severe negative alcohol-related health and psychosocial consequences compared to males (26–29). In addition to vulnerability in adolescence, there are also important gender differences in the impact of adolescent BD on later functioning in adulthood. Notably, females are more likely to experience a more rapid and severe progression from BD to addiction, a phenomenon known as “telescoping” (26). Moreover, while boys who stop abusing alcohol after adolescence are similar to men without any history of alcohol abuse (30), girls who stop abusing alcohol after adolescence continue to differ from women without a history of alcohol abuse in areas of illegal drug use, antisocial behavior, and mental health problems (31). Although prevalence rates of AUD are lower in women compared to men, women with AUD are more likely to experience more negative alcohol-related consequences (31).

In the present review, we will first review some of the literature on gender differences in neurobiological risk factors that predispose an adolescent or emerging adult to engage in BD, given developmental differences between males and females (32, 33). We will also review gender differences in alcohol sensitivity as well as differences in reward neurocircuitry and neurobiological processes in learning and memory that explain differences in risk for BD and response to BD. We will then review some of the literature on gender differences in psychiatric comorbidity among adolescents and emerging adults and the association between this comorbidity and BD. This is especially relevant since 60% of substance-using adolescents have a comorbid psychiatric diagnosis (34). Next, we will review the role of gender in social/peer influences during adolescence and emerging adulthood and how this may influence binge-drinking behavior. Lastly, we summarize findings from existing prevention and intervention research on adolescent and emerging adult BD and important gender considerations in prevention and intervention.

## Method

**Figure 1:**
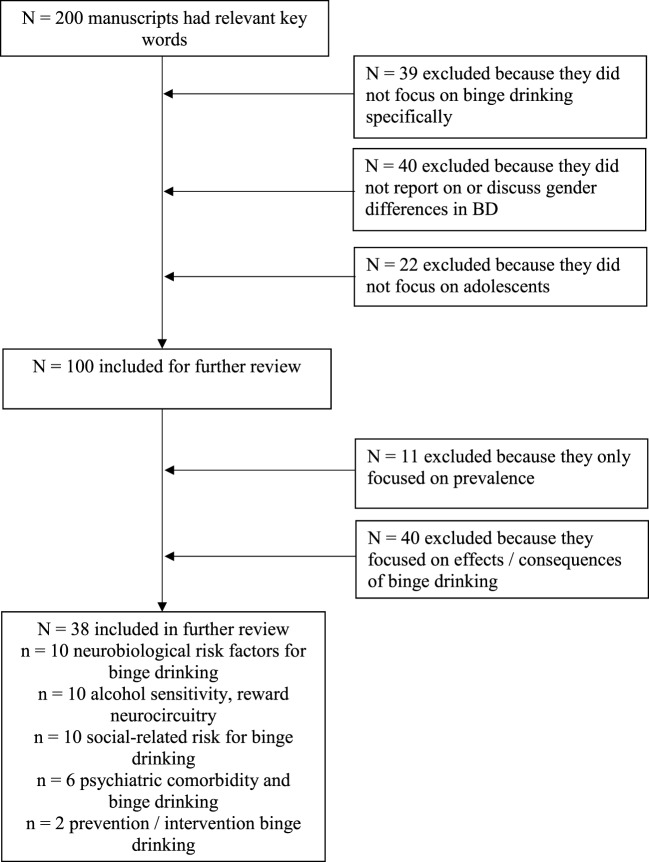
Literature search results. Studies included in this count were specific to all criteria. Given limited studies, discussion also includes relevant studies that pertain to alcohol use and substance use more generally.

An extensive literature search was conducted using MEDLINE/PubMed and Academic Search Premier to identify peer-reviewed publications on adolescent and emerging adult BD published since 2000. There are various definitions for BD across the literature (1, 2, 35) and, thus, we included the literature that defined BD broadly as consuming a large alcohol quantity per drinking occasion (as defined by the WHO, NIAAA, and SAMHSA; 1). For instance, the NIAAA defines BD as consuming at least 4 or 5 (women or men, respectively) drinks in approximately 2 h and achieving a blood alcohol concentration (BAC) of at least 80 mg% (4). In general, all of these definitions include intoxication as a hallmark sign. Thus, we considered literature that defined BD by any of these definitions. We first conducted a broad search using terms for (1) BD, (2) adolescence or emerging adult, and (3) gender/sex to identify all articles that highlighted gender differences in BD. Articles that (1) did not focus on adolescents or emerging adults (age range 13–24); (2) did not consider gender/sex; and (3) did not pertain to BD as defined by either the NIAAA, WHO, or SAMHSA (as described earlier) were excluded. Furthermore, only articles that pertained to risk factors for BD, and not effects or consequences of BD, were selected. We reviewed only articles that reported on gender differences and pertained to (1) social influences, (2) neurobiological and biological aspects of BD risk, (3) psychiatric or mental health symptoms and BD risk, and (4) intervention and prevention for BD (see Figure 1). Annotated bibliographic searches of relevant review articles and/or books were also conducted.

## Results

Figure 1 presents results from the literature search. The initial search yielded a number of studies that focused on gender differences in adolescent BD regarding differential effects and consequences of BD across males and females [see Ref. (36–39) for reviews]. Furthermore, a number of studies reported on BD prevention and intervention, but few focused on gender differences in BD treatment. Therefore, in the sections that follow, we report on identified literature but also incorporate findings from other studies related to problem alcohol use in order to inform potential gender differences in these areas.

### Neurobiological Processes and Risk for BD

Adolescence is a crucial stage of development during which addiction becomes a prominent public health concern (40–46). In the following section, we review literature on adolescents’ unique vulnerability to BD. We first summarize evidence for the role of alcohol sensitivity and reward neurocircuitry in BD during adolescence and highlight gender differences in these processes. We then review the role of adolescent neurobehavioral development in BD as well as important gender differences in development that differentially influence males’ and females’ risk for BD (see Table 1 for overview of studies and findings).

**Table 1:** Overview of studies on gender differences in alcohol sensitivity, neurobiological development, and risk for internalizing and externalizing disorders.

Study	Sample	Binge drinking (BD) measure	Findings
Marco et al. (47)	28 M, 28 F Wistar adolescent rats (PND 0–46)	–	M: increased expression of endocannabinoid-associated genes in frontal cortex following early life stress F: decreased expression of endocannabinoid-associated genes in frontal cortex following early life stress

Koss et al. (48)	5 M, 8 F adolescent rats (PND 20, 35, 90)	–	Both M and F showed decrease in dentritic spines in mfPFC over 90 days postnatal (adulthood); F only showed decrease in basilar dentrites between puberty (P35) and adulthood (P90)

Raznahan et al. (49)	306 F, 312 M (aged 5–25)	–	Sexual dimorphic subcortical maturation F: reach earlier peak volume in striatum (age 12.1 vs. 14.7), thalamus (age 13.8 vs. 17.4) cortex (8 vs. 9.1) M: reach earlier peak volume in pallidum (age 7.7 vs. 9.5)

Blanton et al. (50)	40 F, 32 M community sample (aged 8–18)	–	F: smaller left amygdala (AMY) volume associated with better emotional control M: larger left AMY volume associated with better emotional control

Burghy et al. (51)	28 F, 29 M (age M = 18.44, SD = 0.19)	–	F only: early life stress (retrospective report) predicted increased cortisol levels and decreased AMY-vmPFC connectivity 14 years later F only: AMY-vmPFC connectivity associated with depressive symptoms

Shih et al. (52)	414 M, 402 F community sample (M = 15.2, SD = 0.29)	–	F: higher levels of total and interpersonal stress F: more likely to become depressed in response to stress

Shulman et al. (53)	4,052 F, 4,218 M (aged 10–25)	–	M: higher sensation seeking and lower impulse control vs. F

Adan et al. (54)	60 M, 80 F college students (aged 18–25, M = 21.33)	BD group: at least one past month BDE (30 M, 40 F)	M: binge drinkers characterized by higher sensation seeking F: binge drinkers characterized by higher neuroticism-anxiety

Barnes et al. (55)	9,168 F, 9,542 M 7th–12th graders (aged 12–18, M = 15.1)	Past-year frequency BDE	M stronger relationship between BD and delinquency vs. F

Kuntsche and Müller (56)	1,015 M, 639 F fifth to seventh grade students (aged 11–14, M = 12.5)	–	M: more likely to endorse alcohol enhancement motives F: more likely to endorse coping motives for drinking

Hussong et al. (57)	206 F, 233 M (232 children of alcoholics; T1 aged 10–16, M = 12.7)	Past-year frequency BDE	M only: externalizing symptoms related to increase BDE over 3 years

Chassin et al. (13)	236 M, 210 F (238 children of alcoholics, 208 controls; T1 aged 10–16, M = 13.22)	Past-year frequency BDE (BD groups based on onset and frequency: early-heavy *n* = 93, late-moderate *n* = 134, infrequent *n* = 43, non-BD *n* = 176)	M only: externalizing disorders and low depression related to early-heavy BD F only: depression related to infrequent BD (vs. non-BD)

McGue et al. (58)	Sample 1:625 F, 607 M children of alcoholics Sample 2: 323 F twin pairs (201 MZ) 318 M twin pair (215 MZ)	–	Sample 1: Lifetime externalizing disorders higher in M vs. F children of alcoholics Sample 2: M only: Genetic factors underlying disinhibitory psychopathology related to increased risk of early alcohol use

Danielsson et al. (59)	578 M, 644 F seventh grade students (T1 age 13)	Past-year frequency BDE	M = F: seventh grade BD and smoking predicted ninth grade BD F only: heavy drinking linked to bullying and peer stress

Edwards et al. (60)	7,268 M, 6,709 F community cohort (age T1 M = 12.8, T2 M = 18.66)	AUDIT	F only: longitudinal increase in depressive symptoms between age 12–17 linked to increases in problem alcohol use at age 18

Needham (61)	5,738 F, 5,089 M (T1 age M = 15.3, SD = 1.6)	Past-year frequency BDE	M = F: longitudinal bidirectional relationship between depression and BD

Walsh et al. (62)	1,808 F NSA-R sample (aged 12–17, M = 14.5)	Past-year frequency BDE	In F sexual violence incidents linked to acute increase in BD around the time of the incident

Stevens et al. (63)	274 M, 104 F enrolled in SU treatment (age M = 15.75, SD = 1.03)	GAIN substance use assessment and diagnostic tool	F only: higher levels traumatic stress associated with more severe substance use problems

Foster et al. (31)	636 F community sample (aged 17–29)	DSM-III-R AUD, adolescent onset (AO) desisting *n* = 33, AO persisting *n* = 14, young adult (YA) onset *n* = 43, YA persisting *n* = 27	F: with adolescent-onset AUD greater psychopathology and psychosocial impairment F: with past adolescent AUD still showed impairment in adulthood despite “recovery”

Rohsenow et al. (64)	202 F, 200 M college students (aged 21–24, M = 21.4)	TLFB mean weekly drink quantity	M: lower sensitivity to alcohol effects vs. F based on scores on Self-Rating Effects of Alcohol form

Heath et al. (65)	2,818 F (190 genetic AUD risk), 1,766 M (182 genetic AUD risk), community sample	Maximum alcohol consumption/occasion	M: with genetic AUD risk lower alcohol sensitivity vs. M without AUD risk F: with genetic AUD risk reported more frequent BD vs. F without genetic AUD risk

Bell et al. (66)	12 F (7 peri-adolescent), 12 M (7 peri-adolescent) alcohol-preferring (P) rats (PND 30)	Ethanol intake	F: adolescents more ethanol intake vs. M adolescents M: adults more ethanol intake vs. F adults

Dhaher et al. (67)	Ethanol naïve high alcohol drinking (HAD-1 and HAD-2) rats (PND 21–24)	Ethanol intake, BD = ethanol licking binges	M > F: ethanol consumption and BD (ethanol licking binges) M HAD-2: consistent ethanol intake over 30 days vs. F HAD-2 increasing ethanol intake over 30 days

Schramm-Sapyta et al. (68)	144 M and F peri-adolescent CD Sprague-Dawley rats (PND 21)	Ethanol intake	M = F: no differences in ethanol-conditioned taste aversion

*Only studies highlighting gender differences are presented. PND = postnatal days. BDE = binge drinking episodes, defined as 4/5 or more drinks for females/males per occasion*.

#### Sensitivity to Alcohol during Adolescence

##### Basic Research

The fact that binge ethanol drinking occurs mostly in adolescents and emerging adults is due, at least in part, to the fact that individuals are affected bi-phasically by ethanol in an age-dependent manner (66–69). More specifically, adolescents, compared to adults, show greater sensitivity to lower doses of alcohol, which are perceived as positive and rewarding (e.g., behavioral and autonomic activation), and lower sensitivity to higher doses of alcohol, which are perceived as aversive (e.g., motor ataxia) (44, 45, 70–74). It is believed that this bi-phasic sensitivity in turn not only increases adolescents’ risk for BD but also puts adolescent binge drinkers at increased risk for developing alcohol dependence later in adulthood.

**Figure 2:**
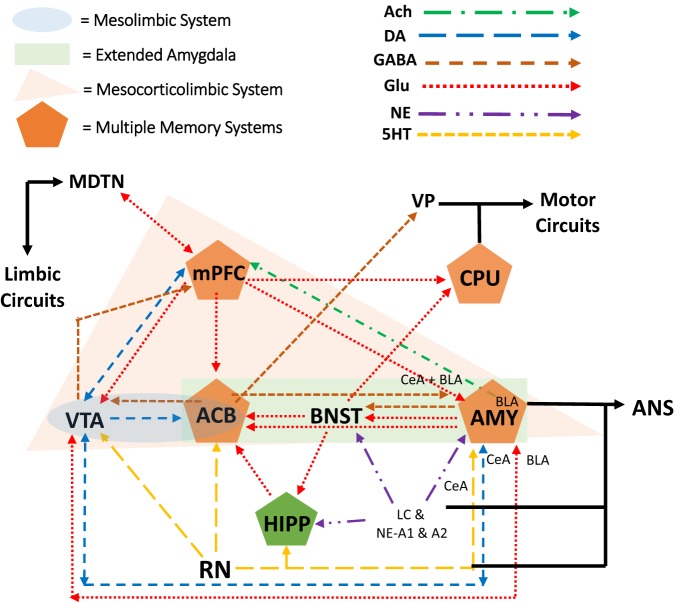
The mesocorticolimbic dopamine reward neurocircuitry mediates orientation toward and acquisition of rewards (e.g., alcohol). At the core of the system are dopamine projections from the ventral tegmental area (VTA), of the midbrain/mesencephalon, to the nucleus accumbens (ACB; i.e., ventral striatum of the limbic circuit). As part of the reward neurocircuit, the nucleus ACB receives dopaminergic projections from the VTA and mediates the intoxicating and euphoric effects of ethanol as well as conditioning of these rewarding effects (i.e., learning and memory). The extended amygdala (AMY) includes nuclei of the AMY, the bed nucleus of the stria terminalis (BNST), and the nucleus ACB shell. The prefrontal cortex (PFC) has reciprocal projections with all of these brain regions while integrating this information with that of other brain regions as well (84, 85). Within the PFC, the medial portions are considered part of the limbic circuit (mPFC). This limbic circuit is associated with the Papez circuit that has been modified to include other brain regions as well (86). Essentially, (a) nuclei of the AMY receive sensory input from the periphery while sending input to the peripheral autonomic nervous system (ANS), (b) the AMY sends and receives information, in part through the stria terminalis, from the septum and hypothalamus, (c) the septum sends and receives information, in part through the fornix, from the hippocampus (HIPP), (d) the HIPP, in turn, sends projections to the hypothalamic mammillary bodies *via* the fornix, (e) the mammillary bodies, in turn, project to the anterior thalamus and mediodorsal thalamic nucleus, which (f) project to the cingulate gyrus and medial PFC (mPFC), and which (g) project back to the entorhinal cortex and HIPP [for recent discussions on the relationship with addiction see Ref. (70, 87–90); Pariyadath et al. (89); Renteria et al. (90)].

One system largely involved in alcohol sensitivity is the mesocorticolimbic system, which is also the reward neurocircuity system (see Figure 2 for explanation of mesocorticolimbic system). Importantly, binge-like alcohol use leads to increases in mesolimbic dopamine and glutamate, which are associated with the development of alcohol dependence (75, 76). For example, animal studies have shown that adolescent binge-like alcohol exposure results in increased ethanol intake and preference later in adulthood as well as a prolonged ethanol-induced increase in mesocorticolimbic dopamine and tolerance to ethanol-induced increases in mesocorticolimbic glutamate during adulthood (77). This finding of altered dopaminergic activity in the adult mesocorticolimbic reward neurocircuit following adolescent binge-like ethanol exposure has been replicated many times (78–80). Furthermore, adolescents also show less sensitivity to withdrawal symptoms following BD, which through negative reinforcement may exacerbate binge-like behavior (81, 82). For example, there is evidence that adolescent binge ethanol exposure followed by protracted withdrawal resulted in a lower ethanol-withdrawal-associated decrease in mesocorticolimbic dopamine than that observed in similarly treated adult rats (83).

With respect to gender differences, there is evidence for diff-erences in mesocorticolimbic activity, which may lead to differences in binge-like alcohol use. For example, stimulant-induced increases in nucleus accumbens dopamine are lower in female rodents compared with their male counterparts (91–93). Clinically, men show a greater mesolimbic dopamine response than women (27, 94), which in part demonstrates males’ greater sensitivity to the rewarding effects of alcohol. The caudate nucleus, also called the dorsal striatum (caudate–putamen or CPU in rodents, Figure 2), mediates habit learning and perseverative behavior, both of which characterize loss-of-control drinking. Estradiol in the dorsal lateral striatum (lateral portions of the caudate nucleus) mediates, in part, stimulant-induced behavioral responses as well as escalation and reinstatement of drug taking behavior (27). These estradiol effects on stimulant-induced and -taking behavior were seen in ovariectomized female rats, but not male rats (91, 95). Importantly, progesterone treatment can reduce these estrogenic effects in female rats as well as reducing stimulant intake in women, but not men (96–98).

In addition to sensitivity and reward, the mesocorticolimbic system is also involved in learning and memory, which are dynamic processes that influence BD. Animal studies have shown unique sex differences in the neurobiological processes of learning and memory. In a study examining the acquisition of an operant response for sucrose, it was found that both adult and adolescent female rats acquired the response quicker than their male counterparts (99). Moreover, these authors reported that after 1 week of training, adolescent female rats responded more than adolescent male rats, whereas adult female and male rats did not differ in their number of responses or reinforcers. Exercise has been shown to decrease ethanol intake during adolescence and appears to have a greater beneficial effect in adult women vs. men (100, 101). In addition, exercise has been shown to facilitate adult neurogenesis in the parahippocampal region of the brain, which has also been implicated in enhanced learning and is disrupted by drugs of abuse including alcohol (102, 103). Thus, it is interesting to note that voluntary exercise during adolescence reduces ethanol intake and preference to a greater extent in female vs. male high ethanol-consuming C57BL/6J mice (104). This is particularly relevant since the hippocampus (HIPP) is vulnerable to ethanol-associated damage, with evidence that adolescents may be more sensitive to this effect than adults.

As noted above, estradiol activity in the lateral caudate nucleus mediates stimulant-induced and -taking behavior, which can be disrupted by progesterone treatment (27, 91). This is important since the caudate nucleus mediates habit formation and is implicated in later stages of the addiction/dependence cycle. Within the multiple memory systems and mesocorticolimbic reward neurocircuitry, endocannabinoid activity modulates emotion and anxiety as well as learning and memory [see Ref. (105) for their roles in addiction]. Given a role for early life stress in vulnerability for addiction and associated behaviors (discussed later), it is noteworthy that the maternal deprivation model of this disorder leads to increased expression of several endocannabinoid-associated genes in the frontal cortex, but not the HIPP, of male rats; whereas the opposite is seen in female rats (47). Early life stress also affects neuroimmune activity, with this effect implicated in adolescent addiction vulnerability (106, 107). Thus, it is noteworthy that during adolescence female rats display greater microglial activation than their male counterparts, suggesting a more adaptive immune system in females during adolescence (107).

##### Clinical Research

While much of the research on alcohol sensitivity, reward, and learning has utilized animal models, evidence for adolescents’ greater sensitivity to alcohol has also been shown in human studies. For example, one study examined college seniors over 4 years and found that hangover insensitivity was significantly correlated with intoxication insensitivity and future alcohol problems, even after controlling for demographic variables (64). With respect to gender differences, experimental studies have shown that that males will drink more alcohol when available and also reach higher BAC’s compared to females (108). In humans, adolescent females appear to be more sensitive to the negative effects of alcohol and experience them at lower doses (65), while males may be more sensitive to the rewarding effects. These differences in effects emerge around the time of puberty and, thus, it is hypothesized that hormone-related changes across males and females are in part responsible (108). While this may be a protective factor for adolescent females (109), they are also more likely to progress more rapidly to addiction than males, due to “telescoping” (110). Still, research on gender differences in risk for BD to dependence trajectories specifically is lacking. As we will discuss next, what may be more important are gender differences in neurobiological-related development that may differentially influence trajectories of risk for BD among males and females.

#### Gender Differences in Neurobehavioral Development

In addition to differences in alcohol sensitivity, reward circuitry, and neurobiological processes of learning and memory, there are also gender differences in development that may differentially influence males’ and females’ risk for BD. For example, females undergo many neurobiological changes earlier than males, and this is in part related to the earlier onset of puberty in females (111, 112). According to the dual systems model, although the striatum matures more quickly than the prefrontal cortex (PFC) in females, it is also suggested that females undergo more extensive maturation in the PFC compared to the striatum in both humans (49, 113) and animals (48, 114). This sex-specific trajectory highlights how females develop greater levels of inhibitory control and lower peak levels of sensation seeking compared to males (53).

In addition to gender differences related to inhibitory control and sensation seeking, the triadic model hypothesizes that there are also gender differences in the development of the amygdala (AMY) as well as differences in connectivity between the PFC and AMY, which influence emotional control (115). In particular, the triadic model posits that development in the AMY and connectivity between the AMY and the PFC may have a greater influence on emotional functioning in females compared to males (33, 50, 51). While, to date, few studies have longitudinally examined the effects of these preclinically assessed neurobiological processes on later risk for BD, we do know that emotion regulation, inhibitory control, and sensation seeking have been linked to BD (54). Thus, these unique neurobiological trajectories in development may manifest as different risk paths to BD among males and females. In the next section, we review literature on the link between psychiatric issues and BD, in particular highlighting distinct risk phenotypes across adolescent males and females.

### Psychiatric Comorbidity and BD

Adolescence is a vulnerable period for developing psychiatric issues (116), in part due to developmental-related brain changes that occur during adolescence (117, 118). The link between psychiatric disorders and substance use is also well established, and it is estimated that up to 60% of adolescents with substance use disorders also meet criteria for another psychiatric disorder (119). Furthermore, the sex-specific neurobiological changes that occur during adolescent development put males and females at differential risk for internalizing and externalizing disorders. For example, gender differences in the development of the AMY as well as connections between the AMY and PFC may increase females’ vulnerability to anxiety and depression (51), while males’ higher peak levels of sensation seeking and slower development of impulse control leaves them more vulnerable to externalizing symptoms (53). Furthermore, gender differences in neurobiological development that occur during adolescence also lead to gender differences in vulnerability to stress and differences in how males and females respond to stress (120). In animal models, protracted stress leads to depressive-like behaviors in females but not males (121). In humans, interpersonal stress is more closely linked to cortisol stress response and internalizing symptoms in female compared to male adolescents (52). Taken together, this highlights how these differences in development and in turn risk for psychiatric issues may beget unique BD risk profiles for males and females.

#### Externalizing Disorders and BD

The link between externalizing symptoms (including behavioral disinhibition, impulsivity, sensation seeking, and defiant behaviors) and substance use has been well documented in the literature (21, 122). As discussed previously, males consistently exhibit higher levels of sensation seeking and behavioral disinhibition throughout development, while females show greater inhibitory control (53). Thus, this externalizing risk phenotype for substance use appears to be more prominent in adolescent boys compared to girls (21). For example, in a recent study of college students, male binge drinkers were characterized by their higher scores on impulsivity and sensation seeking compared to non-BD males, and this pattern was not seen in females (54). Another study also found stronger associations between delinquency and BD in males only (55). Adolescent males are also more likely to report drinking for positive reinforcing effects as well as sensation and risk seeking (26, 56).

There is also evidence that environmental and genetic origins underlying associations between externalizing symptoms and substance use differ by gender. For example, one study found externalizing symptoms mediated the relationship between problematic alcohol use and parental alcoholism in males, but not in females (57). In another study of children of alcoholics, early BD was related to externalizing disorders in boys, but not in girls (13). In another similar study examining children of alcoholics, genetic factors associated with disinhibition and externalizing symptoms were predictive of early drinking for boys only; for girls in the study, environmental risk factors were more closely linked to alcohol initiation (58). Thus, given adolescent males’ higher levels of sensation seeking and lower inhibitory control, and evidence for males’ unique vulnerability to genetic factors underlying the link between externalizing symptoms and BD, it is not surprising that this externalizing risk phenotype for BD and other problem alcohol use is more prominent in males.

#### Internalizing Disorders and BD

Internalizing symptoms, including depression and anxiety, have also been linked to BD (54). As explained above, there are important gender differences in risk for developing internalizing disorders that occur with pubertal development, with girls being twice as likely to develop anxiety and depression compared to boys (117). In addition to the higher rates of internalizing disorders in females, females are also more vulnerable to stress compared to boys (120). Even in animal studies, adolescent female rats exhibited depressive-like behavior following stress, while male rats did not experience depressive-like symptoms (52).

As such, in contrast to males, females’ substance use risk profile is better characterized by internalizing symptoms, such as anxiety, depression, stress vulnerability, and other negative mood symptoms. For example, among a study of college students, female binge drinkers were characterized by higher scores on neuroticism-anxiety compared to non-BD females, while male binge drinkers were better categorized by traits related to sensation seeking and impulse control (54). In addition to females’ heightened vulnerability to stress, females are even more likely to engage in BD in response to stress (123). For example, one Swedish study found that peer bullying and other risk factors had a greater effect on drinking in females than in males (59). Similarly, adolescent girls who abuse alcohol are more likely to have experienced a high level of stressful life events and exhibit post-traumatic stress symptoms, but this is not seen in boys (26).

Females’ higher vulnerability for internalizing disorders also increases their risk for addiction, in part, due to self-medicating tendencies (28). For example, one study found that among females only, greater increases in depression symptoms were also linked to greater increases in problem alcohol use and BD over time (60). Similarly, other studies have shown longitudinal bidirectional relationships between BD and depressive symptoms across adolescence that are particularly strong for females (61). Moreover, among females who began drinking in adolescence, those who continued drinking in adulthood showed high levels of depression during adolescence relative to those who stopped abusing alcohol (31). In addition to females’ greater vulnerability to stress and internalizing symptoms, females may also be more prone to BD following trauma.

#### Trauma and BD

Exposure to potentially traumatic events—such as physical assault or abuse, sexual assault or abuse, and witnessed violence in the home or community—is common in adolescence, with approximately two-thirds of youth reporting exposure to one or more events (124, 125). Trauma exposure has been linked to increased risk of BD and problematic alcohol use, with evidence indicating higher rates of BD among adolescents exposed to childhood maltreatment (126) and greater risk for problematic alcohol use among adolescents exposed to assault and other forms of violence (127). In addition, adolescents exposed to multiple types of victimization are more likely to experience more alcohol abuse (128) than peers who experience fewer victimization types. Hazardous drinking can also increase risk for future trauma and victimization (129, 130). BD also can co-occur with traumatic experiences, in particular, sexual victimization (i.e., drug/alcohol-facilitated and incapacitated sexual assault).

While both male and female adolescents experience sexual victimization, adolescent girls are at heightened vulnerability to sexual assault (131). A recent study of adolescent girls aged 12–17 found that girls who reported drug/alcohol-facilitated and incapacitated sexual assault were more likely to report past-year alcohol abuse than girls with other types of assault or no assault (132). Similarly, sexual victimization predicted acute increases in BD in a national sample of adolescent girls, although victimization did not predict overall escalation of BD over time (62).

Evidence suggests that girls may also be more likely to engage in BD and experience more negative psychological sequelae as a result of trauma experience compared to boys (63). There is evidence that child abuse and neglect predicts later problem drinking for girls, but not boys (133), and that girls (but not boys) who start abusing alcohol during adolescence are more likely to have experienced early traumatic stress (31). Taken together, in addition to females’ increased vulnerability to stress and increased likelihood of BD in response to acute stress, females are also more vulnerable to binge drink in response to more prolonged stress as a result of trauma.

Taken together, this recent research suggests that male and female adolescents exhibit unique BD risk phenotypes: while boys exhibit the traditional externalizing risk phenotype, girls’ risk phenotype is characterized by “internalizing” symptoms, such as high stress reactivity, and the presence of mood disorders and internalizing symptoms (54). These gender differences are related to gender differences in adolescent neural development and are also consistent with findings that adolescent males drink to enhance positive mood states while females drink to avoid negative mood states (134). These findings also highlight the importance of considering trauma in BD, and furthermore, gender differences in males and females’ vulnerability to BD following trauma and stress. Given girls’ increased vulnerability to stress and higher stress reactivity, it is not surprising that the link between trauma exposure and BD is particularly salient in girls.

### Social Influences on BD in Adolescence and Emerging Adulthood

From a social and environmental perspective, across many cultures, adolescence is considered a period of self-exploration and experimentation when individuals start to gain more independence and autonomy from adult caregivers (Table 2). First, this increased autonomy—in combination with developmental-related changes in reward-seeking and decision-making (43–45, 112)—puts adolescents in a vulnerable position of experimentation with less supervision which can result in risky behaviors, such as BD. Second, peer relationships become more important and social influences are prominent during adolescence (135), and are also a key risk factor for alcohol use (136). A number of social-related influences, including social norms, peer pressure, and peer affiliation, have all been shown to influence BD and other alcohol use behaviors (137–139).

**Table 2:** Overview of studies highlighting gender differences in social influences on binge drinking (BD) among adolescents.

Study	Sample	BD measure	Findings
Jalling et al. (140)	85 F, 77 M high school students (aged 12–18, M = 15.09)	AUDIT; frequency BDE	M = F: perceptions of peer BD influenced BD M: parent-report externalizing behavior predicted BD F: social problems predicted BD

Brooks-Russell et al. (141)	1,212 F, 950 M 10th and 11th grade students	Past month frequency alcohol use	M > F: descriptive drinking norms mediated relationship b/w drinking with peers and alcohol use

Zarzar et al. (142)	437 M, 454 F public and private school students(aged 15–19)	Frequency BDE	F only: BD associated with being “close with” school peers vs. church peers (friendship network)

Franca et al. (143)	570 F, 156 M college students (age M = 21.19)	Frequency BDE	M = F: BD associated with overestimation of peer drinking M > F: BD frequency

Griffin et al. (144)	1060 F, 888 M seventh grade students (*n* = 3 schools)	Alcohol quantity/occasion	M > F: reported friends with pro-drinking attitudes M < F: peer drinking norms (i.e., F reported higher peer drink norms)

Elek et al. (145)	4,030 M and F seventh grade students	Past month quantity and frequency alcohol use	Relationship b/w personal drinking norms and substance use stronger for M vs. F M vs. F: descriptive drinking norms stronger predictor of lifetime alcohol use for F vs. M

Hong et al. (146)	731 M, 875 F ninth grade students	Past 2-week frequency BDE	7^th^ grade descriptive norms greater effect on predicting ninth grade BD in M vs. F

Lewis and Neighbors (147)	115 M, 111 F college students (age M = 19.85, SD = 2.39)	Past 3-month frequency BDE	M = F: overestimate same-sex peers’ drinking F > M: same-sex drinking norms stronger relationship with BD in F vs. M

De Visser and McConnell (148)	503 F, 228 M college students (aged 18–25, M = 19.8)	Past month frequency BDE	M > F: intentions of getting drunk F: beliefs in traditional gender roles associated with less BD and drinking intentions M: no relationship between gender role beliefs and alcohol use

Clinkinbeard and Barnum (149)	6,265 F, 4459 M college students (aged 18–25, M = 22.03)	Past 2-week frequency BDE	M > F: BD frequency M only: endorsement of feminine qualities associated with less BD and alcohol-related consequences M = F: endorsement of dominant masculine traits associated with more frequent BD and alcohol-related consequences F only: general masculine traits associated with less alcohol-related consequences

Young et al. (150)	42 F college students (aged 18–22)	Past 2-week frequency BDE	All F reported pressure to BD in order to “impress” male peers

Vetter-O’Hagen, et al. (151)	32 M and F Sprague-Dawley rats (PND 26)	Ethanol intake	M > F: M consumed more ethanol relative to body weight M < F: M less sensitive to aversive alcohol effects when in presence of peer (i.e., social context)

*Only studies highlighting gender differences are presented. BDE = binge drinking episodes, defined as 4/5 or more drinks for females/males per occasion*.

With respect to gender differences on the impact of social influence on behavior, there is even a link between sex-specific brain development and social behavior (109). For one, there is some evidence that girls are more sensitive and vulnerable to social influences, such as peer pressure and peer affiliation compared to boys (24). Association with other drinking peers is particularly influential on BD (140), and some studies have found that drinking peers are a greater risk for BD among females compared to males (59, 141). For example, one study of Brazilian high school students found that peer affiliation was more closely related to BD for girls compared to boys; more specifically, girls who reported being closer to school-based friends vs. family or church friends were more likely to binge drink, and this relationship was not seen among males (142). Furthermore, some studies have shown that adolescent girls are more likely to report drinking in order to obtain peer approval compared to boys (24).

Social norms regarding drinking, or rather, individuals’ perceptions of peers and others’ BD, also influence one’s own drinking behavior. Descriptive norms refer to beliefs about the prevalence of BD among peers while injunctive norms pertain to the perceived social pressure to conform and engage in BD with other peers (137). Social drinking norms are largely dependent on cultural context, and although the majority of studies have examined social drinking norms using US college samples (137), there are some studies that have examined this phenomenon in other areas across Europe (18, 143, 152, 153). There is a pattern across findings that boys are more likely to endorse more permissive or pro-drinking norms (injunctive norms) and perceive higher prevalence rates of BD (descriptive norms) compared to girls (137, 144). However, findings are mixed as to the influence of social norms on actual BD, with some evidence that girls are more influenced by social norms compared to boys (145), and other evidence that social norms are more influential on boys’ BD (146). Thus, further research regarding differential influences of peer norms on BD is warranted.

Along the same lines as drinking norms, gender norms and gender stereotypes are also important to consider in BD (147, 154, 155). Across cultures, there is a double standard for drinking, such that among males, BD is considered more socially acceptable and masculine, while females are often more likely to be judged negatively for BD, as it is seen as less feminine (148, 156). Thus in this way, gender stereotypes may reinforce and perpetuate BD for males (148, 156), while for females, the negative outlook of BD may be a protective factor against BD (149, 156). Still, another study of college females found that females who engaged in more frequent BD did so as a means to feel more equal to their male peers and as a way to impress their male peers (150). Thus, this largely depends on one’s identification with gender roles as well as their motives for BD.

Animal literature has also shown sex differences in adolescent social drinking behavior. Among a study of adolescent Sprague-Dawley rats, adolescent males consumed more ethanol than females when they were in the presence of other peers, and furthermore, males were less sensitive to ethanol’s aversive properties when in the presence of a peer (151). Similarly, in another study of adolescent rats, males consumed more ethanol when in social situations compared to when alone, while females consumed more ethanol when alone; however, there were differential effects across females based on social anxiety-like behavior (157). Female rats with high levels of social anxiety-like behavior had higher ethanol intake in social vs. isolated situations. These findings from animal models (44, 70, 78, 158) suggest that social situations and influences may be more influential on males’ BD behavior. Taken together, these differences in social influences are likely to influence drinking behavior and, therefore, should be addressed in prevention and intervention, and in fact recent research has focused on gender-specific interventions for girls that are based on social learning theory, which is discussed below (see section below; see also sections for sex differences in neurobiological processes of learning and memory).

### Gender Considerations in BD Prevention and Intervention

While there is extensive literature on treating problem alcohol use and AUDs in adults and adolescents, less research has focused on the importance of treating BD in adolescents. One issue is that due to a lack of discrepancy in the literature over BD vs. other alcohol-related problems, treatment literature often does not differentiate target populations, which is important since binge drinkers are a unique typology (1). Psychosocial interventions are recommended as the first-line treatment for alcohol and substance use disorders more generally (34), and cognitive-behavioral skills training and motivational enhancement therapy are the recommended evidence-based strategies [as the most promising evidence-based strategies to target problem drinking (159)]. A few recent literature reviews have summarized existing evidence of the effectiveness of randomized controlled trials of these treatments for binge and other problem drinking for adolescents and college students [see Ref. (160–164) for reviews]. Briefly, interventions that incorporate skills-building, motivational, and personalized normative feedback components have been successful in reducing BD and other problem alcohol use. One limitation noted in these literature reviews and based on the present literature search is the lack of studies’ reports of findings across gender. Thus, in the next sections, we highlight gender considerations in BD intervention and prevention based on findings from the literature discussed previously and other important findings for gender considerations in substance use treatment more broadly (see Table 3 for overview of studies and findings).

**Table 3:** Overview of studies highlighting gender differences in substance use prevention and intervention.

Study	Sample	Binge drinking (BD) measure	Intervention	Main finding
Todd and Mullan (165)	122 F college students (aged 17–25, M = 19.0)	Past 2-week BDE frequency	Mere measurement model (MME, *n* = 40) vs. prototype willingness model (PWM, *n* = 40) vs. control (*n* = 40)	MME group less alcohol vs. control group PWM no effect on BD

Longshore et al. (166)	608 F, 774 M seventh graders (T1)/ninth graders (T2)	Weekly alcohol use	Project ALERT Plus school-based prevention program (*n* = 370) vs. ALERT (*n* = 457) vs. control (*n* = 556)	F: ALERT Plus group decrease in weekly alcohol use at T2 vs. control group M: no difference across ALERT Plus or ALERT vs. control in alcohol use at T2

Schinke et al. (167)	202 daughter–mother dyads community sample (age M = 12.2, SD = 0.95)	Alcohol use frequency in past week, month, year	Mother–daughter computer-based intervention vs. control	Intervention group showed improved alcohol-refusal skills, healthier beliefs about drinking, increased self-efficacy to avoid drinking Intervention group less alcohol use over past week, month, and year

Dunn et al. (168)	19 F, 19 M college students (aged 18–28, M = 21.03)	TLFB 30-day alcohol quantity and frequency	Alcohol expectancy challenge pre and post test	M: decreased alcohol use 30 days following intervention F: no change in alcohol use M only: showed changes in expectancy activation

Hsieh and Hollister (169)	1,462 M, 855 F enrolled in SU treatment as part of Comprehensive Assessment and Treatment Outcome Research (CATOR) sample (aged 12–19)	Measurement of abstinence at follow-up	Non-specific 12-step-based interventions from 24 different residential treatment programs part of CATOR	F: better aftercare and self-help group attendance at 6-month follow-up; more likely to be abstinent at 6-month follow-up M: parental involvement linked to better treatment outcomes

D’Amico (170)	693 M, 813 F middle school students(aged 11–14, M = 12.0)	Past month frequency BDE	–	F: stronger interest in alcohol prevention services M = F past month BDE related to lower intentions to use alcohol prevention services

Stevens et al. (171)	941 M, 266 F from 7 SU treatment programs (age M = 15.75)	Substance frequency and problem index	Non-specific drug treatment programs (*n* = 4 outpatient, 3 residential) across US	F more severe SU and comorbid MH diagnoses at intake M faster rate of change in SU across treatment

Hawke et al. (172)	145 F, 301 M post-SU treatment (aged 12–19)	–	Non-specific “therapeutic community” residential SU treatments across Canada/US	F > M: experience physical and sexual abuse pre and post-treatment M > F: criminal involvement post-treatment

Farabee et al. (173)	805 M, 362 F enrolled in SU treatment as part of Drug Abuse Treatment Outcome Studies for Adolescents sample (aged 11–18, M = 15.7)	–	Non-specific U.S. community-based SU treatment programs (*n* = 6 short-term inpatient, *n* = 8 residential, *n* = 9 outpatient)	M: more likely to enter drug treatment under criminal justice system

Grella et al. (174)	684 M, 308 F enrolled in SU treatment (aged 1–18, M = 15.7)	DSM-III-R AUD criteria	Non-specific U.S. community-based SU treatment programs (*n* = 6 short-term inpatient, *n* = 8 residential, *n* = 9 outpatient)	M = F: adolescents with comorbid disorders more likely to meet criteria for AUD and earlier alcohol initiation vs. adolescents without comorbid diagnoses

Godley et al. (175)	1,550 M, 591 F enrolled in SU treatment (75% aged 15–17)	% days abstinent from alcohol over 90 days	Adolescent community reinforcement approach across 33 U.S. sites	M > F: treatment satisfaction F: higher% days abstinent and more likely to be in recovery at 6-month follow-up M and F: equal gains in recovery

*Only studies highlighting gender differences are presented. BDE = binge drinking episodes, defined as 4/5 or more drinks for females/males per occasion*.

#### Tailoring Treatment

Despite advances in tailoring treatments to address comorbid psychiatric and substance use issues (34), less research has focused on developing gender-specific treatment approaches or identifying gender differences in evidence-based treatments for substance use (176, 177). Over the past few decades, there have been efforts to develop gender-specific treatment programs and focus on issues among adult women; however, less research has focused on adolescent girls in particular (177, 178). Furthermore, many studies do not assess for or report on gender differences in treatment effectiveness (177, 178), and as noted previously, few studies focus intervention for BD specifically. Thus, in the following section, we highlight findings from literature on adolescent substance use treatment more broadly and discuss the potential utility of these findings to inform treatment considerations for BD.

There have been some substance use programs developed to target female adolescents in particular, and these gender-specific programs have been based on social learning and behavior theories (165, 177), which is consistent with the previous discussion that adolescent girls may be more vulnerable to social influences on BD (24). For example, programs focused on social skills training, including teaching assertiveness skills and refusal skills to combat peer pressure, how to develop positive peer networks, and challenge perceptions of the prevalence of alcohol use among peers (e.g., “everyone’s doing it”), are most effective in reducing adolescent girls’ problem alcohol use (166, 177). Furthermore, small group settings may be particularly beneficial for girls, as girls may benefit more from sharing experiences and expressing opinions with others (179). In addition, given girls’ proneness to internalizing symptoms and heightened sensitivity to stress, programs focusing on teaching coping skills and stress and tension reduction techniques may be particularly beneficial (167). For example, the coping skills component of CBT-based treatments are likely particularly beneficial for girls as this may help them to learn healthy and adaptive coping skills to manage stress, negative mood, and other internalizing symptoms that trigger BD.

For boys, given their externalizing risk phenotype, they may benefit more from contingency management techniques that reinforce and reward prosocial behaviors as well as expectancy challenge techniques that challenge their beliefs about the positive effects of drinking (168). Furthermore, the personalized feedback component of MET may be particularly beneficial for boys, given that adolescent boys may be more likely to be “in competition” with or trying to keep up with male peers, given that heavy drinking is seen as more socially acceptable for males and sometimes encouraged, such as in college settings (147). Adolescent boys are more prone to overestimate their peers’ drinking and, thus, challenging these perceptions, such as using personalized feedback, could influence males’ behavior (155). In addition, although gender differences in medication treatment effectiveness among adolescents are unknown (180), among adults, men have better treatment outcomes to pharmacologic treatment for alcohol use than women (181–183). Thus, if these gender differences are similar in adolescents, adolescent males may particularly benefit from pharmacological treatment compared to females.

With respect to gender and parental involvement in treatment, findings are mixed, with some evidence showing more effectiveness of parent involvement in treatment for girls (167) and others showing more effectiveness in boys (169). Among adolescents in residential treatment for substance use, parental involvement in treatment had a significant effect on abstinence at 6-month post-treatment status among boys only; however, treatment characteristics were unknown (169). It may be that the type of parental involvement and family support targeted in treatment should be gender-specific. For example, addressing discipline and rewarding and reinforcing prosocial behaviors may be important for boys given their externalizing risk profile, while for girls, better communication and emotional understanding and support might better target their internalizing risk profile.

#### Gender Differences in Seeking Treatment

There are also important gender differences in treatment seeking. In 2008, only 30% of adolescents who sought substance use treatment were girls (35); however, one study found that girls reported higher intentions to seek treatment for alcohol-related problems (170). One reason for this could be related to treatment referrals. Among adolescents, many substance use treatment referrals come from the juvenile justice system (171). Importantly, boys are more likely to get referred for substance use treatment due to a legal issue (171, 172) or to enter treatment under criminal justice supervision (171, 173). Thus, girls are often not identified as early as boys for needing treatment since the criminal justice system is more likely to identify boys. Girls may be more likely to get referred for treatment or identified from another issue in which BD may be secondary. For example, females seeking substance use treatment in general are twice as likely to be diagnosed with depression (174). Still, girls entering treatment have more severe alcohol problems and higher rates of mental health problems, sexual abuse (171), general health problems (63) and family-related stress (169), while males have more school and legal problems [see Ref. (175) for discussion]. Therefore, girls may also have more severe problems before being identified for treatment which could be detrimental to treatment success. In addition, while not studied in adolescents, among adults, women are less likely to seek treatment due to social stigma, and thus, girls may be less likely to seek treatment due to social stigma as well (28). Due to these differences, further work may need to be done to train and educate health care providers to more effectively screen for and identify BD and other substance use problems in adolescents (184).

#### Treatment Outcomes

There have been mixed findings on treatment outcomes for alcohol and substance use treatment among adolescents. There is some evidence that boys were more likely to become non-drinkers compared to girls following non-specific alcohol use treatment (185), but another study found that girls were more likely to become non-drinkers compared to boys (171). However, one limitation is that these results are based on non-specific treatment across multiple treatment sites (171), thus limiting understanding of specific factors influencing results. One study using data from multiple treatment sites implementing adolescent community reinforcement approach (175) showed similar change rates in substance use problems across boys and girls in treatment but unique course of treatment. Specifically, boys showed quicker improvement in mental health symptoms while girls had more abstinent days from alcohol and were more likely to be in recovery at 6-month follow-up (175). There is also evidence that girls are more likely to utilize social resources and attend after-care and self-help groups such as Alcoholics Anonymous (186), which may lead to better long-term treatment outcomes (169). One consistent finding is that across all adolescents, peer affiliation, school engagement, and parental supervision influence successful treatment in changing adolescents in treatment from binge drinkers to non-binge drinkers (175). Taken together, these mixed findings emphasize the need for further research to determine treatment components that contribute to potential gender differences in outcomes (24, 25). Furthermore, these findings do not address BD in particular, and thus, it is unknown whether these considerations also apply in BD treatment.

Based on the literature review, it is clear that adolescents are a unique, vulnerable population at risk for BD, and that there are important gender differences to consider in treatment. While literature on risk factors and consequences of BD in particular has increased, there is still a gap in the literature on unique considerations in prevention and intervention techniques for BD, as well as in how to effectively target unique differences in psychiatric comorbidity and risks across girls and boy in treatment.

## Conclusion

The review sought to highlight gender differences in risk for BD, focusing on gender differences in (1) adolescent neurobiological development, (2) psychiatric symptoms and the relationship between psychiatric disorders and BD, and (3) social-related risk factors in BD, as well as considerations of these gender differences in BD prevention and intervention. The literature highlights unique vulnerabilities for BD among girls and boys. Developmentally, there are unique risks among boys and girls in relation to BD due to differences in rates of neurobiological changes as well as gender differences in alcohol sensitivity that influence risk for BD. Furthermore, many of these sex-specific neurobiological changes that occur during adolescence also influence differential risk for psychiatric issues among males and females which also influence risk for BD. Notably, while males may be more drawn to BD due to higher levels of sensation seeking and lower inhibitory control, females may be more prone to BD due to their heightened stress reactivity and vulnerability to internalizing symptoms. With respect to social development in adolescence, while development of peer relationships is important for both girls and boys during this developmental period, adolescent girls in particular may be more vulnerable to BD due to social influences. For boys, while peer influence may not be as strong, boys may be at greater risk for BD due to the social gender role norms that it is more socially acceptable and even can be rewarding for boys to drink in excess. These social norms may in turn actually serve as a protective factor in girls as BD does not necessarily align with the feminine stereotype.

These differential risk factors in turn provide important considerations for targeting BD intervention and prevention for females and males. Females may benefit from intervention and prevention that focuses on coping skills training and stress reduction, while males may benefit more from impulse control training and engagement in prosocial activities that fulfill the need for sensation seeking. Regarding social risk factors, while both male and female adolescents would benefit from social skills training, challenging social norms may be more effective for boys while assertiveness skills may be more effective for girls in preventing BD.

## Future Directions

This systematic review highlights two important areas that are in need of further consideration in the literature. The first area is in regard to the necessity of further research on gender-specific risk factors for BD in order to better target at-risk adolescents and also inform prevention for BD. Extensive literature has identified gender differences in the effects of BD on biopsychosocial functioning in adolescents; however, less research has identified risk factors for BD.

There is also extensive literature on theories of adolescent neurobiological development that explain adolescents’ heightened risk for engaging in risk-taking and substance use more generally; however, literature on risk for BD vs. other substance use is lacking. Given evidence that BD is a unique alcohol use typology, more research understanding different mechanisms in the risk process for BD vs. other problem alcohol use vs. other substance use is warranted. Furthermore, given that BD is a hazardous, yet prevalent, developmental phenomenon, more research is needed to better target adolescents that are at risk of developing more severe alcohol use or substance use problems. For example, literature has highlighted the phenomenon of telescoping in women; however, more research on adolescent females and BD is needed.

The second area is the necessity of further research on gender differences in treating and preventing BD among adolescents. For one, many of the randomized controlled trials of BD interventions have focused on college populations, which are a unique group. More research on other adolescent samples, such as younger adolescents, as well as non-college older adolescents, is needed. More importantly, few studies report treatment effects by gender, thus, it is unknown whether there are gender differences in the effectiveness of BD treatment or whether there are gender differences in treatment course or outcomes. Given the increase in BD among adolescent females, as well as the more deleterious effects of alcohol on females, more research in this area is warranted.

## Author Contributions

AD: manuscript concept, writing content across all sections, editing all sections, and references. RB: writing content for introduction, biological developmental risk section, and editing content. ZA: writing content for trauma and binge drinking section, and intervention section. LH: manuscript concept, writing content for biological developmental risk section, editing content across all sections.

## Conflict of Interest Statement

The authors declare that the research was conducted in the absence of any commercial or financial relationships that could be construed as a potential conflict of interest.
